# A Novel Two-Axis Differential Resonant Accelerometer Based on Graphene with Transmission Beams

**DOI:** 10.3390/s22020641

**Published:** 2022-01-14

**Authors:** Yang Xiao, Feng Hu, Yuchen Zhang, Jiaxing Zheng, Shiqiao Qin

**Affiliations:** College of Advanced Interdisciplinary Studies, National University of Defense Technology, Changsha 410073, China; xiaoyang624@nudt.edu.cn (Y.X.); hufeng@nudt.edu.cn (F.H.); z_yc98@nudt.edu.cn (Y.Z.); zhengjiaxing@nudt.edu.cn (J.Z.)

**Keywords:** graphene resonant, biaxial accelerometer, finite element simulation, differential mode

## Abstract

In this paper, a novel two-axis differential resonant accelerometer based on graphene with transmission beams is presented. This accelerometer can not only reduce the cross sensitivity, but also overcome the influence of gravity, realizing fast and accurate measurement of the direction and magnitude of acceleration on the horizontal plane. The simulation results show that the critical buckling acceleration is 460 g, the linear range is 0–89 g, while the differential sensitivity is 50,919 Hz/g, which is generally higher than that of the resonant accelerometer reported previously. Thus, the accelerometer belongs to the ultra-high sensitivity accelerometer. In addition, increasing the length and tension of graphene can obviously increase the critical linear acceleration and critical buckling acceleration with the decreasing sensitivity of the accelerometer. Additionally, the size change of the force transfer structure can significantly affect the detection performance. As the etching accuracy reaches the order of 100 nm, the critical buckling acceleration can reach up to 5 × 10^4^ g, with a sensitivity of 250 Hz/g. To sum up, a feasible design of a biaxial graphene resonant accelerometer is proposed in this work, which provides a theoretical reference for the fabrication of a graphene accelerometer with high precision and stability.

## 1. Introduction

An accelerometer is a sensor that measures acceleration by calculating the inertia force or displacement of proof mass under external acceleration. It is widely used in inertial navigation, attitude measurement, and other scientific and industrial fields [1]. According to the signal type, accelerometers can be divided into capacitive [2], piezoresistive [3], piezoelectric [4], resonant accelerometers, and so on. Among them, the resonant accelerometer measures acceleration based on the highly sensitive response of the resonant frequency of the oscillator to stress. It has distortionless outputs signals, anti-interference ability, good repeatability, and high sensitivity [5].

Reducing the size of the oscillator is of great significance to meet the requirements of a faster response speed, higher accuracy, and wider range of acceleration measurement [6]. The emergence of graphene and other two-dimensional materials paves the way for miniaturizing accelerometers. Graphene has excellent mechanical, electrical, and optical properties [7,8]. The mechanical properties include high strength, high stiffness, low quality, high resonance frequency, and wide tuning ability [9,10], which makes graphene a promising material for the oscillator in accelerometers.

In 2007, J. S. Bunch et al. [11] fabricated and measured the graphene resonator for the first time. Since then, research on the graphene resonator and its applications, such as the resonant accelerometer, has been gradually developed. However, due to the difficulties in device fabrication and detection, the research on the resonant graphene accelerometer is still in the stage of theoretical research and simulation calculation. In 2012, Jeong Won Kang et al. [12] studied a resonant accelerometer based on graphene nanoribbons by means of molecular dynamics simulation. It was found that when the acceleration was in the range of $10^{10}\sim 10^{14}\;$g, the resonant frequency increased linearly with the acceleration on the logarithmic-logarithmic scale. On this basis, they studied the resonant characteristics of the crossroad-type graphene accelerometer. When the acceleration was greater than $10^{13}$ g, the linear relationship between the resonant frequency and acceleration on the logarithmic-logarithmic scale was also measured [13]. Through molecular dynamics simulation, Ki-Ryang Byun et al. [14] also studied a graphene resonant accelerometer with additional mass. The results also showed the linear relationship on the logarithmic-logarithmic scale. When the attached mass increased, the sensitivity to acceleration decreased while the measurement range remained constant. Although the results of the three studies above are similar, the order of acceleration is quite large, which is not consistent with the practical application conditions. Daniel Moreno et al. [15] fabricated resonant graphene accelerometers with suspended silicon proof masses. The devices can detect accelerations of the order of tens of micro-g with a sensitivity of around 1935 Hz/g. In addition, in 2018, Fu-Tao Shi et al. [16] designed a differential graphene resonant accelerometer, which connected one edge of graphene with the proof mass. The axial strain of graphene was changed by the displacement of the proof mass under acceleration, and the acceleration was detected via the frequency shift of graphene. Through finite element simulation, in the range of 0–1000 g, the acceleration sensitivity can reach 21,224 Hz/g, realizing the ultra-sensitive acceleration measurement. Furthermore, researchers from Beijing University of Aeronautics and Astronautics designed the appearance of a biaxial resonant accelerometer but did not study its performance [17].

On these grounds, a novel biaxial graphene resonant accelerometer and its measurement performance were studied in this work. More importantly, in order to avoid non-axial applied force of graphene due to gravity and acceleration in other directions, transmission beams were designed to indirectly connect graphene with the proof mass. Thus, the cross sensitivity between two axes is reduced, realizing the independent measurement on the orthogonal biaxial. At the same time, two graphene resonant beams working in a differential state were designed on each axis, which improves the sensitivity of acceleration measurement. In addition, the measurement performance of the accelerometer was evaluated through finite element simulation. By optimizing the corresponding parameters of graphene and the size of the proof mass and transmission beams, the accelerometer was designed to meet the requirements of a proper measuring range and high sensitivity.

## 2. Model Design and Establishment

### 2.1. Structure of Accelerometer

**Figure 1:**
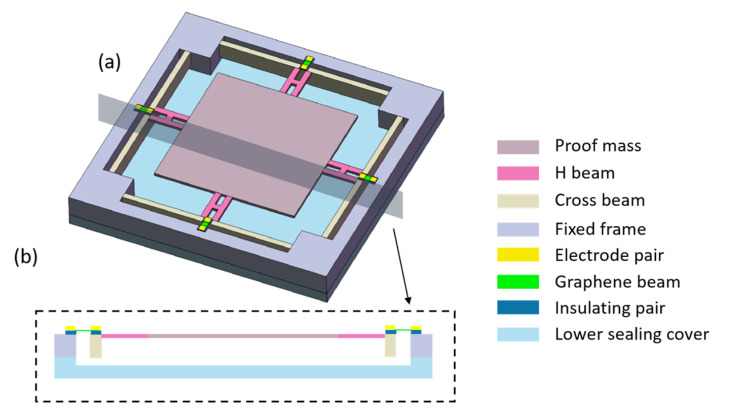
Schematic diagram of accelerometer. (a) Overall structure of accelerometer. (b) Cross section of accelerometer.

As shown in Figure 1a,b, the accelerometer consists of a proof mass, resonant components, and fixed frame with central hollows. The resonant components include transmission beams, electrode pairs, insulator pairs, and graphene bands. The proof mass in the center is supported by four groups of transmission beams and suspended, which overcomes the friction between the fixed frame and the proof mass. Each group of transmission beams consists of an H beam and a crossbeam. One end of the H beam is connected with the center of the crossbeam, while the other end is connected with the proof mass, and the upper surfaces of these two beams are in the same horizontal plane to facilitate etching. The thickness of the crossbeam is usually larger than that of the H beam, so that the lateral displacement of the crossbeam introduced by the gravity of the proof mass and transmission beams is too small to be considered. The main structure of the accelerometer is formed by etching of a double-sided polished silicon wafer and then the main body and sealing covers are closely combined by the bonding process of the silicon wafer [18,19]. The crossbeam, H beam, and the central proof mass are located in a straight line, so that the whole accelerometer is symmetrical along two axes to avoid off-axis disturbance of acceleration. Four pairs of silicon dioxide layers or other insulating layers are covered at the central position on the crossbeam and the fixed frame. Then, four monolayer or few-layer graphene tapes are transferred to the corresponding insulating layers through the micro-nano transfer platform. The electrode pairs are formed at both ends of four graphene tapes by sputtering metal, and the graphene tapes are fixed at the same time. Finally, the accelerometer is sealed in a vacuum environment by bonding with the upper (not drawn) and lower sealing cover and vacuuming [20]. The resonant frequency can be actuated and detected by electrostatic [21] or optical [22] schemes. When using electrostatic excitation and detection schemes, the electrodes at both ends of graphene serve as source and drain, respectively, while the crossbeam is used as gate. All of them are connected with the external detection circuit by setting signal leads on the upper sealing cover. When using the optical excitation and detection schemes, the upper sealing cover is made of glass or other transparent materials.

### 2.2. Working Principle of the Accelerometer

When there is external acceleration, the proof mass is subjected to the inertia force F=ma and transfers it to the H beam. Since the axial stiffness of the H beam is much larger than the tangential stiffness, when axial acceleration exists, the H beam drives the crossbeam with a small transverse deformation under the corresponding axial force, resulting in a small displacement at one end of the graphene belt and leading to a change of the stress and resonant frequency of graphene. However, when there is acceleration in the tangential direction, the H beam has a large deformation in the tangential direction, and the concentrated force transferred to the crossbeam is so small that it can be ignored and hardly influences the stress of the graphene beam. Therefore, the independent measurement of the orthogonal acceleration in the horizontal plane is realized.

**Figure 2:**
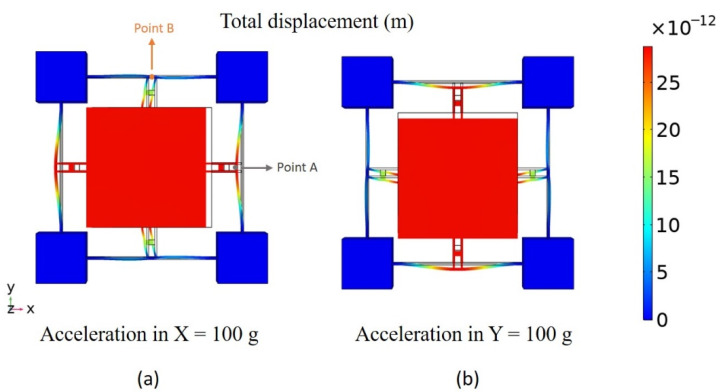
The total displacement diagram of the accelerometer with the acceleration of 100 g along the positive X axis (**a**) and the positive Y axis (**b**), respectively. The fixed frame of the accelerometer is simplified.

Figure 2 shows a three-dimensional diagram of the total displacement in the finite element model with an acceleration of 100 g along the positive X axis and Y axis, where point A and B are the midpoints of the outer edges of the crossbeams along the X axis and Y axis, respectively, corresponding to the midpoints of the fixed edges of the graphene beams. With the acceleration along the X axis, the displacement of point A is much larger than that of point B, which means the combination of H beams and cross beams can effectively reduce the cross sensitivity. It is worth noting that gravity is considered in the model, which meets the requirements of practical application.

**Figure 3:**
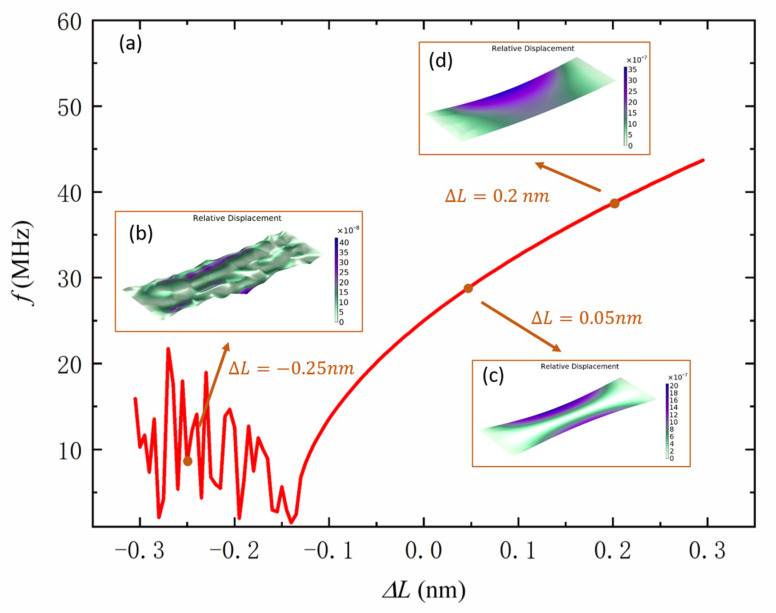
(**a**) Variation of the graphene resonant frequency with the stretching or shrinking length. (**b**) The resonant mode of graphene with a shrinking length of 0.25 nm. (**c**) The resonant mode of graphene with a stretching length of 0.05 nm. (**d**) The resonant mode of graphene with a stretching length of 0.2 nm.

In order to improve the sensitivity of acceleration measurement, a pair of graphene resonant beams working in a differential state are placed on each axis. The inertia force produced by axial acceleration stretches one graphene beam while it shrinks the other at the same time, resulting in reverse resonant frequency shifts of these two graphene beams. Graphene has a fracture strength up to 42 N/m [23], so it can withstand large acceleration without fracture. Figure 3a shows the resonant frequency of a graphene beam with a fixed edge of 1 μm and unfixed edge of 3 μm when the graphene beam is stretched or contracted. When stretched, the resonant frequency rises with the increasing stretching length, and there is a linear region close to the origin. With the further increase of the stretching length, the growth of the resonant frequency becomes nonlinear, and the growth rate decreases continuously. Figure 3c,d shows the resonant modes of graphene beams with a stretching length of 0.05 and 0.2 nm, respectively, and their corresponding strains are $1.6\times 10^{-5}$ and $6.67\times 10^{-5}$. The resonant mode of graphene is symmetrical with weak stretch, but with the increase of the stretching strength, the maximum amplitude appears on the free edge of one side, which is called edge mode [24]. However, the change of the resonant mode does not affect the continuity of the resonant frequency with the stretching length. When the graphene beam shrinks, the resonant frequency decreases with the increasing shrinking length. Additionally, the resonant mode is similar to that in Figure 3c, which is not shown in the figure. With the further increase of the shrinking length, buckling of graphene occurs. At this time, the calculation result of the resonant frequency is imaginary, and its absolute value also changes irregularly. As shown in Figure 3b, the resonant mode is singular as well. The resonant frequency no longer directly reflects the shrinking length, which means the measurement of acceleration is invalid.

## 3. Modeling and Simulation

In this paper, the accelerometer was modeled and simulated by using the finite element simulation software COMSOL Multiphysics [25]. The modeling process of the accelerometer was divided into two parts: displacement calculation and resonance frequency calculation. The first part calculated the displacement of the midpoints on the outer edges of crossbeams when the proof mass was subjected to inertia force, which adopted the steady-state calculation of the solid mechanics module in COMSOL Multiphysics. The second part calculated the resonant frequency of graphene under the corresponding stretching or contraction length calculated in the first part, which was calculated by the steady-eigen frequency calculation of the shell module. Thus, the resonant frequency of graphene with different accelerations was obtained by combining these two parts of calculation. All the calculated results were convergent.

**Figure 4:**
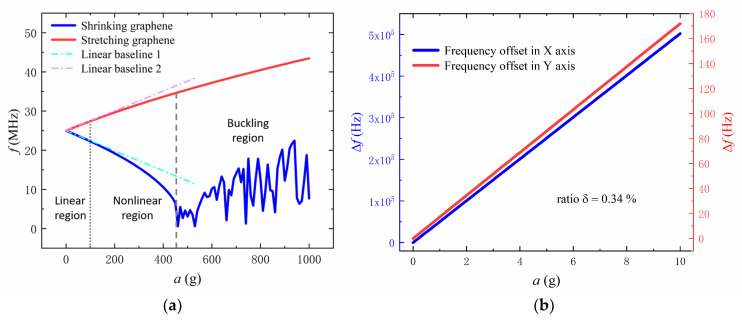
(**a**) The resonant frequency of a pair of differential graphene beams varies with acceleration. Two dashed lines are linear baselines with *a* = 0 as the common origin. (**b**) The resonant frequency offsets of the graphene in the X axis and Y axis vary with the acceleration along the X axis. *δ* is the ratio of the frequency offset in the Y axis to the frequency offset in the X axis.

The initial size of the accelerometer is shown in Table 1. With certain parameters of the accelerometer, quantitative analysis of measurement performance can be performed. Figure 4a shows the change of the resonant frequency of a pair of differential graphene beams corresponding to the acceleration along the same axis. When the acceleration is small, the increase of the resonant frequency of the stretching graphene beam can be regarded as linear, while the resonant frequency of the shrinking graphene beam decreases linearly, which is called the linear region. We define a linear region with a linear deviation of no more than 0.5%. Based on this, the linear range is 0–89 g with an acceleration step of 1 g. Using graphene as a harmonic oscillator will lead to a higher level of scale factor nonlinearity than silicon. The nonlinear scaling factor of a silicon-based accelerometer is usually dozens [26,27], but for shrinking graphene, the nonlinear scaling factor can reach hundreds or even thousands. Therefore, it is very important to further develop the measurement of acceleration in the nonlinear region in order to expand the application range of the graphene accelerometer. In the nonlinear region, with the rise of acceleration, the decreasing rate of shrinking graphene increases, while the increasing rate of stretching graphene decreases. When the acceleration is greater than 460 g, buckling of graphene occurs, which is called the buckling region. Thus, the critical buckling acceleration is 460 g. Additionally, the sensitivity of shrinking graphene in the linear region is 25,591 Hz/g, and that of stretching graphene is 25,328 Hz/g after calculation. Therefore, the differential sensitivity of acceleration is 50,919 Hz/g, which belongs to the ultra-high sensitivity accelerometer [16]. In order to calculate the cross sensitivity of the accelerometer, the resonant offsets of graphene in the X axis and Y axis were calculated, with the acceleration along the X axis. As shown in Figure 4b, the cross sensitivity of the accelerometer is 0.34%. In addition, with acceleration along the X axis, it will also lead to a tiny non-axial displacement of one end of the graphene in the X axis. However, since the displacement is very small compared with the axial displacement, and the sensitivity of the resonant frequency to the non-axial strain is much lower than the axial strain, this displacement can usually be ignored in the calculation (the frequency deviation ∆f/f does not exceed $2\times 10^{-9}$ when the acceleration is within 100 g).

Here, we list the sensitivity, cross sensitivity, and other parameters of the resonant accelerometers in this work and other published work, as shown in Table 2. The sensitivity of graphene-based accelerometers is 100 to 1000 times higher than that of silicon-based accelerometers, while the volume of graphene-based accelerometers is fairly low. For the cross sensitivity, the accelerometer in this work also shows a very low value compared to the silicon-based accelerometers. These results prove that the graphene-based resonant accelerometers have an obviously superior performance, which may make a breakthrough in the application of resonant accelerometers.

**Figure 5:**
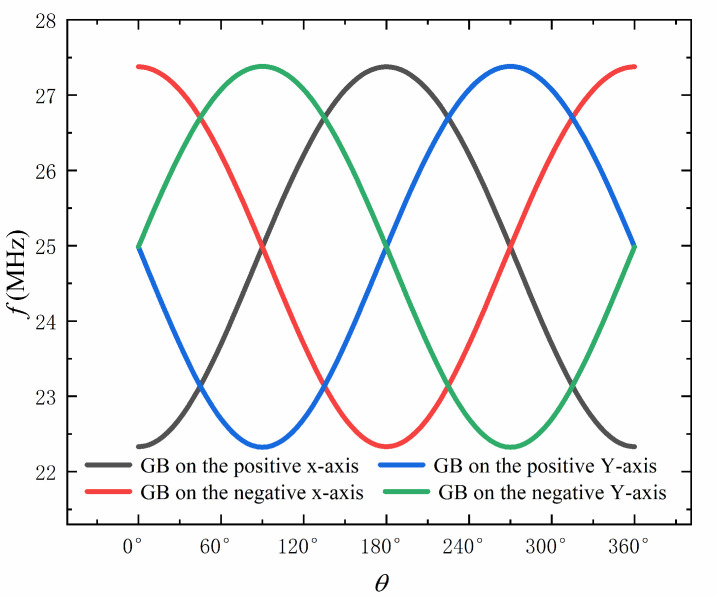
The resonant frequency of the four graphene beams (GBs) in the orthogonal axis varies with the angle between the acceleration and the positive X axis with acceleration of 100 g.

The biaxial accelerometer can also measure the direction of the acceleration in the plane [31]. Since the acceleration in the orthogonal axis is measured independently, the direction of the acceleration can be obtained by vector calculation. Figure 5 shows that the resonant frequencies of the four graphene beams in the orthogonal axis vary with the angle between the acceleration and the positive X axis when the absolute value of acceleration is 100 g. It can be seen that the resonant frequency of these four graphene beams has different trends with the change of the angle; thus, by detecting the resonant frequencies of these four graphene beams, the magnitude and direction of the acceleration in the plane can be obtained quickly and accurately.

## 4. Optimization and Results

### 4.1. Parameter Optimization of Graphene Beam

By adjusting the structure of the accelerometer, the measurement range and sensitivity of the acceleration can change to a great extent but inversely. The structure of the accelerometer can be optimized for different measurement requirements and practice engineering conditions. Two methods are usually used to approach this purpose: adjusting the corresponding parameters of the graphene beam and the size of the force transfer structure, which contains a proof mass and transmission beams.

**Figure 6:**
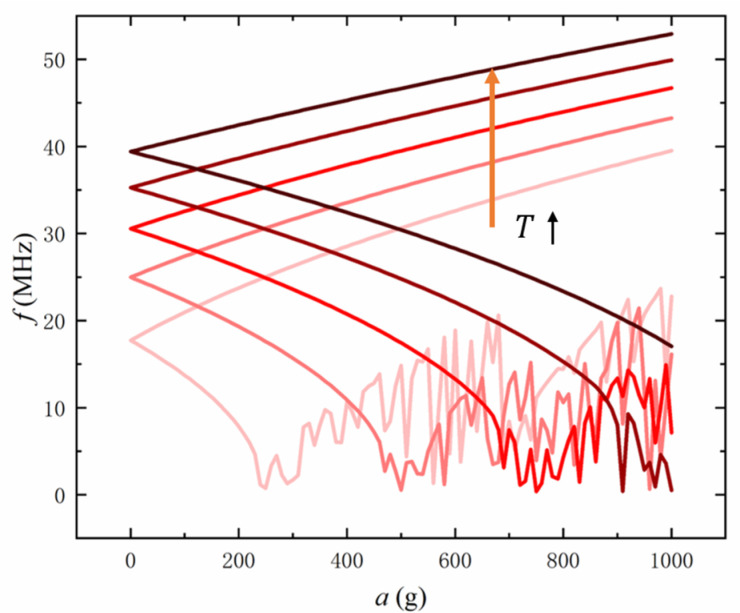
The resonant frequency of stretching and shrinking graphene beams with different tension varies with acceleration, in which the tension from low to high is 0.01, 0.02, 0.03, 0.04, 0.05 N/m, respectively.

Firstly, the influence of the corresponding parameters of graphene on the measurement performance was studied. It is reported that the prepared graphene is generally under the built-in tension. The graphene strain is reported in the order of $10^{-5}\sim 10^{-4}$ [32], and the relationship between strain and tension can be expressed as [33]: T=Et/(1−ν)ε, where E is the Young’s modulus; ν is Poisson’s ratio; t and ε are the thickness and surface strain of graphene, respectively. Thus, we set the studied tension range to T=0.01 N/m ~ 0.05 N/m. Figure 6 shows that, with the increase of tension, the resonance frequency of the graphene beam rises, while the critical buckling acceleration and critical linear acceleration also significantly increase. As the tension rises by five times, the critical buckling acceleration increases more than five times. This result shows that increasing the built-in tension of graphene can meet the application conditions that require a higher measurement range, while reducing the tension can enhance the acceleration measurement sensitivity. The built-in tension is usually introduced during the fabrication process, such as the self-tension produced when graphene adheres to the groove wall by van der Waals force after transfer [34]. Furthermore, the tension can be increased by applying a gate voltage [35] between the graphene beam and the gate, while releasing part of the built-in tension can usually be achieved by current annealing [36].

**Figure 7:**
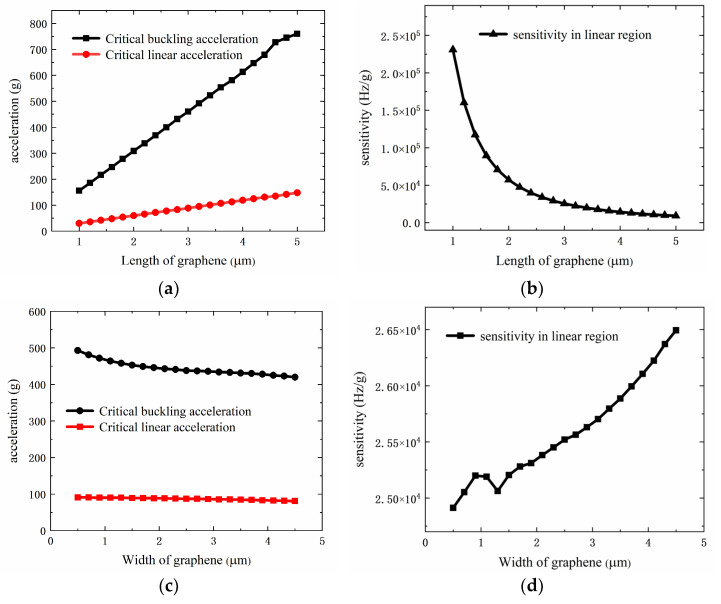
The critical buckling acceleration, critical linear acceleration (**a**), and sensitivity (**b**) of the accelerometer vary with the length of graphene. The critical buckling acceleration, critical linear acceleration (**c**), and sensitivity (**d**) of the accelerometer vary with the width of graphene.

In addition, the effect of the graphene size on the performance of acceleration measurement was also studied. To make the results intuitive, we extracted the critical buckling acceleration and critical linear acceleration from the acceleration–frequency curves, and calculated the acceleration sensitivity of the shrinking graphene beam in the linear region. The variation of the above parameters with the length (free edge) of the graphene beam is shown in Figure 7a,b. With the increase of the length, both the critical buckling acceleration and critical linear acceleration rise approximately linearly, but the increasing rate of the critical linear acceleration is slower than that of the critical buckling acceleration. The sensitivity of acceleration decreases exponentially with the increasing length, so reducing the length of graphene can effectively increase the sensitivity of acceleration measurement. As shown in Figure 7c,d, the width (fixed edge) of graphene has a much weaker effect on measurement performance than the length. Additionally, the variation trend of the acceleration range and sensitivity with the width is opposite to that of the length.

### 4.2. Size Optimization of Force Transfer Structure

**Figure 8:**
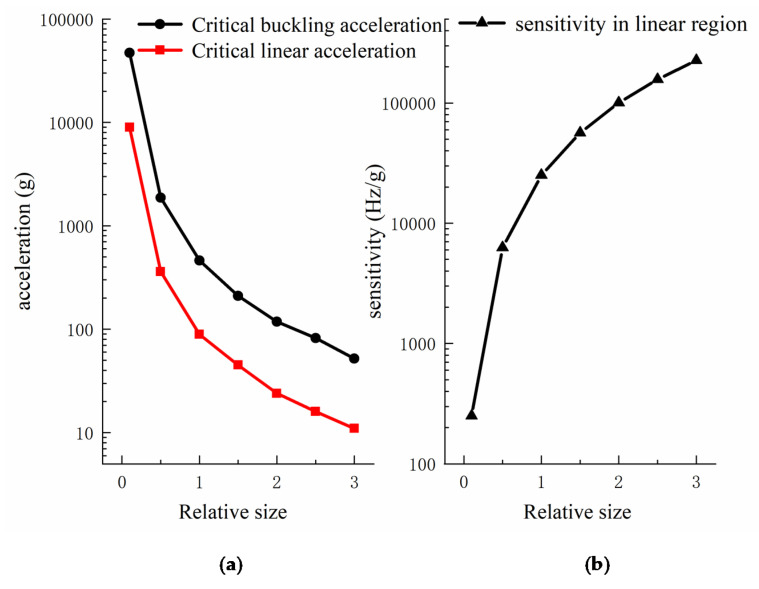
The critical buckling acceleration, critical linear acceleration (**a**), and acceleration sensitivity (**b**) of the accelerometer vary with the relative size of the force transfer structure.

Secondly, the size of the proof mass and transmission beams affects the measurement performance of acceleration by changing the corresponding stretching and shrinking length of the graphene under acceleration. Since the relative size of each structure was designed considering the independence of measurement and avoiding off-axis crosstalk, we only studied the synchronous magnification or reduction of these structures. We studied the measurement performance of the accelerometer in the range of 10 times reduction to 3 times magnification based on the original size, and the results are shown in Figure 8. It was found that the critical buckling acceleration and critical linear acceleration are greatly affected by the size. When the size is reduced by 10 times, the critical buckling acceleration is increased 100 times to nearly $5\times 10^{4}$ g, which can meet the requirements of acceleration measurement in extreme environments, such as high-speed impact [37]. At this time, the sensitivity is on the order of hundreds Hz/g. However, the etching accuracy should reach 100 nm or even 10 nm, which requires a high level of fabrication technology and can be achieved by electron beam lithography or extreme ultra-violet lithography [38]. When the relative size is enlarged by 2 times and 3 times, the corresponding acceleration sensitivity increases by nearly 4 times and 9 times, respectively, which is approximately quadratic increasing. Therefore, changing the size of the force transfer structure is an effective method to improve the acceleration measurement performance.

## 5. Summary

In this work, a novel two-axis differential resonant accelerometer based on graphene was proposed, in which the design of the transmission beams enables the accelerometer to not only reduce the cross sensitivity and realize independent measurement on the orthogonal axis, but also overcome the influence of gravity on acceleration measurement. Therefore, in practical application, the accelerometer can quickly and accurately detect the magnitude and direction of acceleration. In addition, two graphene resonant beams work in the differential state on each axis, which improves the measurement sensitivity of acceleration. The accelerometer was modeled using finite element simulation software COMSOL Multiphysics, and the modeling process can be divided into the displacement calculation part and the resonance frequency calculation part, which completely simulates the working process of the accelerometer. The calculation results showed that the critical buckling acceleration is 460 g, and the linear range is 0–89 g. When the accelerometer works in the differential mode, its measurement sensitivity is 50,919 Hz/g, belonging to the ultra-high sensitivity accelerometer. In addition, the range and sensitivity of the accelerometer can be adjusted by optimizing the corresponding parameters of graphene and the size of the force transfer structure. For example, increasing the length and tension of graphene can significantly increase the critical linear acceleration and critical buckling acceleration, but the sensitivity will also decrease. Furthermore, when the size of the force transfer structure is reduced by 10 times synchronously, which requires the etching accuracy to reach 100 nm, the critical buckling acceleration range of the accelerometer will be close to $5\times 10^{4}$ g. Additionally, when the size is enlarged, the acceleration sensitivity will increase quadratically. In summary, the graphene biaxial resonant accelerometer introduced in this paper lays a theoretical foundation for the fabrication of a resonant graphene accelerometer with high sensitivity and stability. However, there are still many challenges in the fabrication and measurement of graphene-based resonant accelerometers, such as the low-quality factor at room temperature, difficulties in controlling graphene prestress, and so on. Therefore, there is an urgent need to explore the preparation process and integrated technology for the application of graphene resonant accelerometers.

## Figures and Tables

**Table 1:** Initial size of the accelerometer.

Structure	Size (Length× Width×Thicness)
Proof mass	60 μm×60 μm×2.5 μm
H beam (the long)	15 μm×1 μm×2.5 μm
H beam (the short)	3 μm×1 μm×2.5 μm
Crossbeam	66 μm×1 μm×9 μm
The suspended part of graphene	3 μm×1 μm×0.34 nm

**Table 2:** The performance parameters of different resonant accelerometers.

Reference	Materials	Dimension	Volume (μm×μm×μm)	Sensitivity (Hz/g)	Cross Sensitivity
Caspani et al. [28]	Silicon-based	biaxial	720×720×15	250	5%
Ding et al. [29]	Silicon-based	biaxial	1900×1900×25	275	3.4%
Yang et al. [30]	Silicon-based	biaxial	7500×7500×70	52.57 (X-axis) 51.64 (Y-axis)	1.08% (X-axis) 1.33% (Y-axis)
Shi et al. [16]	Graphene-based	uniaxial	≈70×70×10	21,224	-
Morenoet et al. [15]	Graphene-based	uniaxial	5×5×16.4 (proof mass)	1935	-
This work	Graphene-based	biaxial	≈120×120×10	50,919	0.034%

## Data Availability

The data are included in the main text.
